# Editorial: *In silico* Methods for Drug Design and Discovery

**DOI:** 10.3389/fchem.2020.00612

**Published:** 2020-08-07

**Authors:** Simone Brogi, Teodorico Castro Ramalho, Kamil Kuca, José L. Medina-Franco, Marian Valko

**Affiliations:** ^1^Department of Pharmacy, University of Pisa, Pisa, Italy; ^2^Laboratory of Molecular Modeling, Chemistry Department, Federal University of Lavras, Lavras, Brazil; ^3^Department of Chemistry, Faculty of Science, University of Hradec Kralove, Kralove, Czechia; ^4^DIFACQUIM Research Group, Department of Pharmacy, School of Chemistry, Universidad Nacional Autónoma de México, Mexico City, Mexico; ^5^Faculty of Chemical and Food Technology, Slovak Technical University, Bratislava, Slovakia

**Keywords:** chemoinformatics, computational chemistry, computational methods in medicinal chemistry, computer-aided-drug design, drug discovery, molecular modeling, web-servers

Computer-aided drug design (CADD) methodologies are playing an ever-increasing role in drug discovery that are critical in the cost-effective identification of promising drug candidates. These computational methods are relevant in limiting the use of animal models in pharmacological research, for aiding the rational design of novel and safe drug candidates, and for repositioning marketed drugs, supporting medicinal chemists and pharmacologists during the drug discovery trajectory. Within this field of research, we launched a Research Topic in *Frontiers in Chemistry* in March 2019 entitled “*In silico Methods for Drug Design and Discovery*,” which involved two sections of the journal: Medicinal and Pharmaceutical Chemistry and Theoretical and Computational Chemistry. For the reasons mentioned, this Research Topic attracted the attention of scientists and received a large number of submitted manuscripts. Among them 27 Original Research articles, five Review articles, and two Perspective articles have been published within the Research Topic. The Original Research articles cover most of the topics in CADD, reporting advanced *in silico* methods in drug discovery, while the Review articles offer a point of view of some computer-driven techniques applied to drug research. Finally, the Perspective articles provide a vision of specific computational approaches with an outlook in the modern era of CADD.

Regarding the Original Research articles, two of them are related to innovative approaches concerning ADMET properties of the molecules. In particular, de Bruyn Kops et al. reported the development and validation of GLORY, an innovative tool for predicting the metabolism of molecules, identifying chemical structures of metabolites formed by cytochrome P450 enzyme family (CYPs). The mentioned software combines two main ideas: a literature-based pool of CYP-mediated reaction rules and the site of metabolism (SoM) prediction. This approach is relevant since a tool for the *in silico* prediction of the metabolism of xenobiotic compounds can offer key information for developing novel chemical entities with improved metabolic stability (i.e., cosmetics, drugs, agrochemicals). The GLORY web-server version is accessible at https://acm.zbh.uni-hamburg.de/glory/ (de Bruyn Kops et al.). Montanari et al. described a computational approach for predicting potential toxicity of molecules taking into account transporter proteins. These latter proteins, expressed in the liver, are crucial in drug pharmacokinetics and are important constituents of the physiological bile flow and their inhibition could be relevant to the drug-induced liver toxicity. Using a comprehensive analysis of the publicly available data, a set of classification models was developed for predicting the inhibition of the transport for a set of liver transporters deemed relevant by different regulatory agencies. The models were computationally validated demonstrating an ability to predict the interaction profile of small molecules with liver transporters. These computational tools can assist medicinal chemists and toxicologists in prioritizing compounds at the initial steps of the development of drug candidates. The models are freely available as a web-service at https://livertox.univie.ac.at (Montanari et al.). Another work, regarding the pharmacological profiles of molecules, was presented by Sidorov et al.. They investigated the possibility to predict synergism of cancer drug combinations using NCI-ALMANAC data. This topic is of extreme interest since drug combinations could represent a promising strategy for treating cancer. The authors described an *in silico* approach to investigate drug combination synergy by exploiting the largest available dataset reporting synergism of anticancer drugs (NCI-ALMANAC, with over 290,000 synergy determinations). Two machine learning (ML) procedures, Random Forest (RF), and Extreme Gradient Boosting (XGBoost), were employed on the selected dataset. The assessment of these computational tools indicated that the prediction of the synergy of undisclosed drug combinations is a feasible task. Accordingly, by using these kind of models it will be possible to significantly reduce the number of *in vitro* tests, by evaluating *in silico* which of the selected combinations are expected to be synergistic (Sidorov et al.).

A relevant number of papers are focused on different ligand- and structure-based approaches or on a combination thereof to identify promising molecules for a given target. Accordingly, Velázquez-Libera et al. described a combined structure- and ligand-based approach for investigating the structural requirements governing the affinity of a series of molecules for the human Sigma1 receptor (S1R). This receptor represents a valuable drug target for treating neuropsychological disorders. The authors discovered an effective S1R agonist namely RC-33 as a promising neuroprotective agent. In the paper presented in this Research Topic, the authors computationally investigated the interactions of RC-33 and its novel derivatives within the S1R active site. To this end, different *in silico* techniques [docking, interaction fingerprints, and receptor-guided alignment three-dimensional quantitative structure-activity relationship (3D-QSAR)] were applied for investigating a potential mechanism of action of the developed compounds. The presented data could be useful for designing novel S1R modulators (Velázquez-Libera et al.). Wu et al. also described a combination of different computational procedures (*de novo* protein structure prediction and ligand-protein interaction simulation) to investigate the structural requirements of compounds governing the affinity for the *h*SK2/calmodulin complex. The authors developed a homology model of SK2/calmodulin in order to predict potential binding sites. The ligand-protein interaction, using a series of computational procedures, was then investigated. The obtained results confirmed that the combination of different *in silico* techniques could facilitate the drug discovery process (Wu et al.). Furthermore, some computational approaches, including 3D-QSAR, molecular docking, virtual screening (VS), ADME prediction, and molecular dynamics (MD), were used by Chen et al. to identify some HIV-1 non-nucleoside reverse transcriptase (RT) inhibitors (NNRTIs). Starting from a novel series of dihydrofuro[3,4-d]pyrimidine (DHPY) related compounds, endowed with antiviral activity, a computational investigation was performed employing 52 DHPYs. By applying sequential *in silico* methods, nine promising compounds were identified. These hit compounds could represent novel potential HIV-1 NNRTIs. Chen et al.. For identifying novel BCL-2 inhibitors from the Specs -SC- database, Tutumlu et al. employed multistep screening and filtering methods combining structure- and ligand-based techniques. The mentioned database was screened using a computational tool called “*cancer-QSAR”* and 26 toxicity QSAR models. The resulting non-toxic compounds were selected for two different target-driven approaches: (a) a molecular docking approach was applied to rank compounds considering their docking scores. Top-ranked compounds were employed in extensive MD simulations (100 ns) and biological assays; (b) the retrieved top-docking poses of each compound, derived from the subset selected by QSAR studies, were submitted to short MD simulations (1 ns), calculating their binding energies using the molecular mechanics generalized Born surface area (MM/GBSA) technique. By following this scheme, seven molecules were tested against different cancer cell lines. Four molecules were found to be able to reduce the proliferation of cancer cells, behaving as pro-apoptotic agents (Tutumlu et al.). The study performed by do Carmo et al. was also focused on BCL-2 and potential ligands based on a phenothiazine scaffold. The authors investigated some phenothiazines derivatives for their pro-apoptotic profile, performing an *in silico* study to relate their structures with their biological activities. By employing molecular docking simulation coupled to MD, the main interactions between compounds and the active site of the selected protein were highlighted. Notably, through these computational studies, the inhibition of BCL-2 by phenothiazines allowed for rationalizing the apoptosis-inducing effect on tumor cells (do Carmo et al.).

Naveja and Medina-Franco described a computational approach for selecting lead compounds from large datasets of chemical entities, acquired by high-throughput screening (HTS). They introduced the Constellation Plots as a general method for merging diverse and complementary molecular representations, to enhance the info contained in a visual representation and analysis of chemical space. This approach combines a sub-structure-based representation and classification of molecules with a “classical” coordinate-based representation of chemical space. A characteristic result of the mentioned technique is that organizing the molecules in analog series leads to the formation of groups of compounds, also known as “constellations,” in chemical space. Notably, this proposed method is useful in identifying, for example, insightful and “bright” Structure-Activity Relationships (StARs) in chemical space that are simple to interpret. The authors applied the developed method on two datasets of DNA methyltransferases (DNMTs) and AKT1 inhibitors (Naveja and Medina-Franco). Alberca et al. reported a computational approach that allowed for the repurposing of old drugs as antimalarial agents. The authors developed and experimentally validated a collection of ligand-based models that are able to identify falcipain-2 inhibitors. These models were used in a VS campaign, using two different databases (DrugBank and Sweetlead). The authors identified four potential hits to submit for biological evaluation. Among them, two drugs (odanacatib and methacycline) were confirmed as falcipain-2 inhibitors. Methacycline was found to be a non-competitive inhibitor of falcipain-2. Furthermore, the effects of both drugs on falcipain-2 hemoglobinase activity and on the growth of *P. falciparum* have been investigated (Alberca et al.). Baillif et al. reported a computational study, using a public dataset of compound-induced transcriptomic, for predicting the potential activity of compounds against 69 drug targets. The authors investigated the performances of the ML models constructed with transcriptomics data, with the computational tools generated by Morgan fingerprints. Active molecules against a given target could display comparable signatures in one or multiple cell lines, independent of the similarity in chemical structure, among the selected active chemical entities. For 25% of the tasks, RF computational tools employing transcriptomics signatures showed similar or better performances than those created by Morgan fingerprints. Compound-induced transcriptomic data offers a good chance for predicting targets based on cell response similarity, allowing to overcome the chemical space limitation of QSAR models (Baillif et al.). Shi et al. computationally investigated the SAR of some inhibitors of the dimerization process of PD-L1 by elucidating their potential binding and unbinding mechanism, using classical MD and metadynamics simulations. The contact analysis, R-group based QSAR analysis, and molecular docking provided additional insights about the SAR of these compounds. Accordingly, the outcomes of this research can be useful for optimizing compounds targeting PD-L1 (Shi et al.). Liu et al. introduced two methods for improving the selection of active molecules by using similarity information of all compounds. One technique ranks a molecule considering its highest z-score as an alternative of its highest Tanimoto index, while the other method ranks compounds by calculating an aggregated score taking into account their Tanimoto similarity related to all identified active and inactive molecules. These evaluations, performed using datasets available from PubChem, belonging to over 20 HTS studies, suggested that both approaches accomplished a ~10% higher Boltzmann-enhanced discrimination of receiver operating characteristic (BEDROC) score, compared to the classical approaches. Interestingly, the presented methods could offer an enhancement in early recognition of lead compounds during VS campaigns (Liu et al.). Lima et al. presented a computational approach for finding multi-kinase inhibitors against *Plasmodium falciparum* calcium-dependent protein kinases 1/ 4 (CDPK1 and CDPK4, respectively) and protein kinase 6 (PK6), in order to select novel multi-target compounds as antimalarials. By using shape-based and ML models, employing chemical databases of drug-like compounds, the authors identified 10 hit compounds to submit for biological evaluation. Among them, LabMol-171, LabMol-172, and LabMol-181 behaved as nanomolar antiplasmodial agents. Furthermore, LabMol-171 and LabMol-181 also inhibited *P. berghei* ookinete development, representing novel transmission-blocking agents (Lima et al.). Bühlmann and Reymond reported an approach to address the limitation of the GDB17 database (166.4 billion molecules), which contains numerous molecules that are too complex to synthesize. To this end, the authors developed the GDBChEMBL database, a small set of GDB17, which consists of 10 million molecules identified by means of the calculation of their ChEMBL-likeness score (CLscore). This subset contains compounds with higher synthetic accessibility, maintaining a comprehensive coverage of chemical space distinctive of the GDB17 database. GDBChEMBL is downloadable from http://gdb.unibe.ch; interactive chemical space map: http://faerun.gdb.tools (Bühlmann and Reymond).

Sirous et al. developed and experimentally validated an *in silico* procedure useful for hit-to-lead optimization. In particular, from micromolar HIV integrase (HIV IN) inhibitors, the authors described a computational workflow based on an *in silico* structure-based combinatorial library designing technique. The mentioned methodology is useful for combining the design of a combinatorial library and side-chain hopping with Quantum Polarized Ligand Docking (QPLD) and MD simulations. This method indicated the most valuable decorations for a promising scaffold. From this final set of optimized molecules, three representative compounds were synthesized and evaluated by *in vitro* tests. Among them, one compound was found to be an effective inhibitor of HIV IN in the low nanomolar range. Moreover, the biological characterization of the molecule showed that this compound is able to inhibit HIV-1 replication and HIV-1 IN strand transfer activity, with potency comparable to that found for Raltegravir (Sirous et al.). Ferreira et al. presented an article describing the development of cyclic imides as inhibitors of cruzain, a validated drug target of *Trypanosoma cruzi*. By using a micromolar-range cruzain inhibitor, the *in silico* optimization scheme led to the development of a non-toxic inhibitor of *T. cruzi* intracellular amastigotes in the nanomolar-range. By following the mentioned procedure, the authors identified a protocol useful for the rational design of novel trypanocidal agents targeting the cruzain enzyme (Ferreira et al.). Pallante et al. proposed a computational approach based on different *in silico* techniques such as homology modeling, molecular docking, and MD for investigating the interactions between several novel colchicine derivatives and tubulin isotype βIII. These derivatives were screened and ranked considering their binding affinity and conformational stability in the colchicine binding site. This study could be extremely relevant for rationally designing novel colchicine-based compounds as effective anticancer agents (Pallante et al.). Pavlin et al. focused their research article on the application and experimental validation of a VS protocol to identify small molecules that are able to target a particular variant of estrogen receptor alpha (ERα Y357S) that confers endocrine resistance, disease relapse, and increased mortality rates in patients affected by ER-positive breast cancer. By applying a VS procedure for screening different commercial databases, the authors identified five compounds active on recurrent Y537S ERα polymorphism in MCF7, and MDA-MB-231 breast cancer cell lines. Among the identified compounds, one of them showed selectivity for Y537S ERα, exhibiting no toxicity against breast cells. Remarkably, 4.5 μs of biased and unbiased MD was used for investigating the structural, thermodynamics, and the kinetics of these active ligands against wild type and diverse ERα variants (Y537S, Y537N, D538G). The information provided by the mentioned study could be relevant for discovering mutant specific drug-candidates for improving breast cancer therapies (Pavlin et al.). Quan et al. investigated novel quinoline derivatives as P-glycoprotein (P-gp) inhibitors useful for counteracting the multidrug resistance, which represents a significant cause of cancer treatment failure. Among the mentioned derivatives, YS-7a was proposed as the most promising P-gp inhibitor. YS-7a blocked the P-gp transport without influencing the P-gp expression. Furthermore, YS-7a promoted the ATPase activity of P-gp in a dose-dependent manner. This compound could represent a valid starting point for developing novel derivatives that are able to treat multidrug resistant cancers (Quan et al.).

Michel et al., using different web-servers (DoGSite, FTMap, and CryptoSite) and a commercial tool (Schrödinger's SiteMap), comprehensively predicted ligand binding cavities, druggability scores, and conformationally active regions of the nucleoside diphosphates attached to the sequence-x (NUDIX) hydrolase protein family. Subsequently, a molecular docking study, employing Glide software, was carried out to assess the affinity of a subset of the ZINC FragNow database for the identified potential binding sites. This preliminary dual ranking, of druggable sites within the NUDIX protein family, was then compared with experimental hit rates acquired from biological studies. The detected correlation indicated that the described workflow could represent a valuable protocol for prioritizing targets and for excluding them in VS approaches (Michel et al.). Michel et al. presented a sequence-to-structure-based methodology for predicting drug resistance. The developed workflow produced and compared Molecular Interaction Fields (MIF), mapping the areas of energetically favorable interactions, between numerous chemical probes and the target active site. The technique appears to be appropriate for understanding changes of the three-dimensional structures and the physicochemical environment caused by mutations affecting the target active site. This approach was applied to four datasets of known HIV-1 protease sequences, displaying that it is able to correctly classify resistant and susceptible sequences given as the input. The described study is a novel step for interpreting the influence of genetic variability on the response to HIV 1 treatments (Alves et al.). Sánchez-Tejeda et al. proposed a vector analysis for measuring and defining “multitargeticity.” The research on multi-target drugs could be relevant for identifying therapeutic agents to treat multifaceted diseases. The authors, considering the order and force of a ligand, described two “multi-target” indexes namely, 1 and 2. By combining the mentioned indexes, it is possible to discriminate multi-target drugs. These indexes were used for screening a chemical library of potential ligands that possess an affinity for diverse targets involved in multiple sclerosis. The application of the protocol allowed the identification of 10 molecules that could represent potential lead compounds for developing multi-target drugs (Sanchez-Tejeda et al.). Bissaro et al. applied the SuMD technique to ribonucleotide targets of pharmacological interest. SuMD is a modified MD protocol for accelerating the sampling of molecular recognition steps on a nanosecond timescale. Interestingly, they demonstrated the methodological ability of SuMD to reproduce the binding mode of viral or prokaryotic ribonucleic complexes and artificially engineered aptamers with a remarkable accuracy (Bissaro et al.).

Cavasotto and Aucar proposed a new approach for scoring results obtained from high-throughput docking (HTD) approaches. For better characterizing protein-ligand interactions, the authors proposed a quantum mechanical (QM)-based docking scoring function in order to obtain more accurate HTD results. This novel technique was investigated using 10 different drug targets belonging to various families with diverse binding site features. The output clearly demonstrated that the application of the QM scoring function could improve the performance of HTD methods (Cavasotto and Aucar). Pinto et al. presented a novel screening software, namely, CaverDock. In particular, the authors focused their studies on protein tunnels and channels that could represent promising drug targets. In fact, compounds able to hinder the entrance of substrates or release of products could be considered effective modulators of the biological activity. To this end, the influence of rigid and flexible side-chains on various substrates and inhibitors of seven unrelated drug targets was assessed. The accuracy of the software was evaluated by comparing the data found by CaverDock with experimental results obtained for the heat shock protein 90α. As a final point, CaverDock was used in a VS campaign employing anti-inflammatory and anticancer FDA-approved drugs against two drug targets [CYP450-17A1; leukotriene-A4 hydrolase (LTA4H)/aminopeptidase (AP)]. The analysis of the potential energies of binding and unbinding trajectories allowed for identifying functional tunnels. Accordingly, the presented software is a valuable computational resource useful in VS campaigns. CaverDock is accessible from https://loschmidt.chemi.muni.cz/caverdock/; web https://loschmidt.chemi.muni.cz/caverweb/ (Pinto et al.). Yuan et al. presented LigBuilder-V3, a software for *de novo* multi-target drug design. This computational tool can be useful for rationally designing and optimizing molecules with multi-target profiles. For validating the computational approach, LigBuilder-V3 was employed to design inhibitors that are able to target HIV protease and HIV RT, employing three different approaches. The resulting molecules, assessed by MM/GBSA, behaved as potential inhibitors for the selected drug targets. The software can be found at http://www.pkumdl.cn/ligbuilder3/ (Yuan et al.).

In this Research Topic two Perspective articles have been published. In the first article, Rastelli and Pinzi focused the attention on the need to obtain a valid post-docking analysis in VS campaigns. In fact, nowadays, HTD is a valuable *in silico* methodology extremely useful for rapidly identifying hit compounds for a given target. Unfortunately, HTD has some weaknesses (i.e., approximated scoring functions, limited sampling of ligand-target complexes), making docking outputs inevitably approximate. So, post-docking analyses are required to overcome these mentioned issues. The authors proposed a comprehensive method for the post-docking analysis in VS approaches, developing BEAR (Binding Estimation After Refinement), a post-docking resource this is able to refine docking poses employing MD, and re-scores ligands using (MM/PB(GB)SA). The article provides a rational perspective about the introduction of more accurate refinement and rescoring approaches in VS to provide more reliable results (Rastelli and Pinzi). In the second Perspective article, Vásquez and González Barrios investigated the ligand efficiency (LE) metrics and how to improve it for an efficient and reliable hit identification in CADD. LE is still employed as a decision-making criteria in CADD. Furthermore, in fragment-based drug discovery (FBDD), LE metrics are extremely efficient in selecting promising “core” fragments for optimization. The authors presented a relative group contribution (RGC) model for improving FBDD. Accordingly, this approach could be useful in virtual fragment-based screening studies (Vásquez and González Barrios).

Finally, five Review articles reported a critical discussion about different computational approaches in various CADD fields. Santos et al. discussed the use of Molecular Mechanics (MM) for describing protein-ligand interactions. The authors reported a case study on the halogen bonds (XBs). Although less efficient than hydrogen bonds (HB), XBs were found to be relevant in CADD. XBs are able to improve the affinity and selectivity of a molecule against a potential drug target. Due to the limited ability of MM techniques to describe XBs, the authors indicated that a parametrization of the force-fields equations could be considered for improving the definition of XBs. This review highlighted some suggestions for parametrizing force-fields to accomplish reliable outcomes of complex non-covalent interactions (Santos et al.). Gagic et al. reviewed the *in silico* approaches employed for designing compounds behaving as kinase inhibitors. Kinases are pivotal in designing anticancer agents. They discussed and compared, considering some representative case studies, different methods such as pharmacophore modeling, MD, VS, and molecular docking for the rational design of kinase inhibitors (Gagic et al.). de Souza Neto et al. proposed a Review article for outlining the FBDD strategies exploring numerous computational strategies to apply for fragment-to-lead optimization. They considered potential fragment expansion strategies such as hot spot analysis, druggability prediction, SAR, application of ML/deep learning (DL) models for VS and some *de novo* approaches for suggesting synthesizable novel molecules. Moreover, the authors highlighted some recent case studies in FBDD, and how computational approaches were successfully used for developing lead compounds (de Souza Neto et al.). Maia et al. presented a Review article which reported a comprehensive outlook on the tasks in CADD for performing structure-based VS (SBVS). The authors compared methods and tools for SBVS employed in the modern drug development trajectory (Maia et al.). Thafar et al. discussed some established techniques using artificial intelligence (AI), ML, and DL approaches for identifying drug-target interactions (DTIs) and for predicting drug-target binding affinities (DTBA). This Review article reported an inclusive summary about the computational approaches for predicting DTBA. Notably, this review performed the first inclusive comparison analysis of *in silico* tools focused on DTBA related to AI/ML/DL (Thafar et al.).

In summary, as Guest Editors, we would like to thank all the authors and co-authors for their important contributions to this Research Topic, all the reviewers for their valuable work in evaluating the submitted manuscripts, and the editorial staff of Frontiers for their kind continued assistance. Taken together all these combined efforts allowed for the great success of this Research Topic. We expect this topic to contribute to the advancement of drug design and discovery and believe it serves as a valuable source of information and inspiration to scientists and students. The Research Topic is freely accessible through the following link https://www.frontiersin.org/research-topics/10032/in-silico-methods-for-drug-design-and-discovery.

## Author Contributions

All authors listed have made a substantial, direct and intellectual contribution to the work, and approved it for publication.

## Conflict of Interest

The authors declare that the research was conducted in the absence of any commercial or financial relationships that could be construed as a potential conflict of interest.

